# Pt Catalysts Supported on Ni-N-Doped Carbon Nanotubes for Oxygen Reduction Reaction

**DOI:** 10.3390/ma19112331

**Published:** 2026-06-01

**Authors:** Shuyue Xia, Yilin Yuan, Qinghong Huang, Yuping Wu

**Affiliations:** School of Energy Science and Engineering, Nanjing Tech University, Nanjing 210037, China; xiashuyue0623@163.com (S.X.);

**Keywords:** polyaniline, CNTs, oxygen reduction reaction, catalyst

## Abstract

**Highlights:**

- An ultra-low-Pt-loading Pt/Ni-N-CNT catalyst was successfully developed using carbon nanotubes as templates.
- The PANI-derived N-doped carbon layer uniformly encapsulates the CNT skeleton, ensuring highly dispersed Pt nanoparticles.
- Pt/Ni-N-CNT delivers superior ORR activity in an acidic electrolyte, exhibiting a prominent half-wave potential of 0.846 V (vs. RHE) that outperforms commercial Pt/C (0.81 V vs. RHE).
- The catalyst outperforms commercial Pt/C in both limiting diffusion current density and oxygen reduction kinetics.

**Abstract:**

This study aimed to develop high-performance, ultra-low Pt-loading 2.1 wt% vs. 20 wt% for commercial Pt/C) oxygen reduction reaction (ORR) catalysts. Utilizing carbon nanotubes (CNTs) as templates, a PANI layer was coated onto the surface to serve as a nitrogen-doped anchoring layer for metal species. Physical and structural characterizations demonstrated that the PANI-derived nitrogen-doped carbon layer uniformly encapsulates the CNT skeleton. This architecture not only achieved highly uniform Pt nanoparticle dispersion but also induced strong metal–support electronic interactions via deep-seated Ni atoms, effectively optimizing the electronic structure of the surface Pt. Electrochemical results showed that Pt/Ni-N-CNT delivers superior ORR activity in an acidic electrolyte, with a half-wave potential of 0.846 V (vs. RHE) and limiting diffusion current density outperforming commercial Pt/C (0.81 V vs. RHE), demonstrating excellent oxygen reduction kinetics.

## 1. Introduction

Fuel cells, as highly efficient and clean energy conversion devices, demonstrate immense potential in the field of sustainable energy [1,2]. However, the sluggish kinetics of the cathodic oxygen reduction reaction (ORR) necessitate the use of substantial amounts of noble metal platinum (Pt) catalysts to accelerate reaction rates, which has emerged as a major bottleneck hindering the commercialization and large-scale deployment of fuel cells [3]. Currently, commercial Pt/C catalysts typically require high platinum loading (approximately 20 wt%) [4], which not only escalates costs but also faces challenges related to the scarcity of Pt resources and insufficient catalyst stability [5,6]. Consequently, the development of ORR catalysts with low Pt loading, high activity, and superior stability has become a focal point of current research in the field of electrocatalysis [7,8]. To overcome these limitations and achieve superior electrocatalytic performance, recent advanced studies have demonstrated several effective strategies. These include engineering complex porous or branched Pt-based nanostructures [9], maximizing the noble metal utilization through atomic-level or single-atom alloying configurations [10], and employing nitrogen-enriched porous carbon materials as robust catalyst supports [11].

Carbon nanotubes (CNTs) have been extensively employed as carbon supports for fuel cell catalysts owing to their exceptional electrical conductivity, high specific surface area, and robust chemical stability [12]. Research indicates that the incorporation of heteroatoms, such as nitrogen, into the CNT framework can significantly modulate their electronic structure and surface chemical properties, thereby enhancing oxygen molecule adsorption and catalytic activity [13,14]. Furthermore, when N-CNTs serve as supports for Pt nanoparticles, they can optimize the electronic structure of Pt through metal–support interactions (MSIs), leading to improved Pt utilization efficiency and catalytic performance [15]. Polyaniline (PANI), as a conducting polymer rich in nitrogen and possessing good chemical stability, is an ideal precursor for the synthesis of nitrogen-doped carbon materials [16,17]. Coating a PANI layer onto the surface of CNTs not only provides an abundant nitrogen source but also facilitates the formation of a unique core–shell structure, which increases the specific surface area and active site density of the catalyst [18,19]. Upon high-temperature pyrolysis, nitrogen atoms from PANI can be effectively doped into the CNT skeleton, yielding an M-N-CNT structure with exceptional oxygen reduction reaction (ORR) activity [20]. This PANI-based nitrogen-doping strategy enables uniform distribution and controllable regulation of nitrogen species, providing an ideal pathway for fabricating high-performance non-precious metal or low-Pt-loading catalysts [21,22]. In recent years, constructing Pt-based catalysts with low Pt loading to reduce fuel cell costs has emerged as a significant research direction [23]. Anchoring trace Pt nanoparticles onto nitrogen-doped carbon supports allows for a significant reduction in Pt consumption while maintaining or even enhancing catalytic activity through the synergistic effect between Pt and N-CNT sites [24]. Studies indicate that as Pt loading decreases, electronic interactions at the metal–support interface become increasingly critical, effectively modulating the electronic state of Pt and optimizing the adsorption energy of oxygen species, thereby enhancing the intrinsic ORR activity [25,26].

Against this background, this study synthesized a novel oxygen reduction catalyst with high activity, robust electrochemical stability, and ultra-low Pt loading 2.1 wt% by leveraging polyaniline-coated carbon nanotubes (PANI-CNTs). The novelty of this work resides not merely in the integration of these components, but in the synergistic structural and electronic modulation enabled by our specific synthesis sequence. The PANI-derived nitrogen-doped layer provides a dense array of anchoring sites that physically stabilize the ultra-low amount of Pt nanoparticles, while the deep-seated Ni atoms cooperatively optimize the electronic d-band center of the surface Pt. This dual synergy allows the catalyst to kinetically outperform commercial 20 wt% Pt/C, providing both a theoretical and experimental basis for the design of cost-effective, high-performance fuel cell cathode catalysts.

## 2. Materials and Methods

### 2.1. Material Preparation

#### 2.1.1. Pretreatment of Aniline

Considering that aniline monomers are highly susceptible to oxidation and subsequent discoloration (turning reddish) during long-term storage, decompression distillation was employed for purification and decolorization to ensure the reliability of the synthesis. After this purification process, the collected aniline liquid reverted to a colorless and transparent state. All aniline used in the subsequent preparation steps of this study was pretreated via this vacuum distillation method.

#### 2.1.2. Pretreatment of Carbon Nanotubes (CNTs)

Prior to use, the surfaces of the CNTs were functionalized with a 12 M mixture of HNO_3_ and H_2_SO_4_ in a 7:3 (*v*/*v*) ratio. Specifically, 0.1 g of CNTs was dispersed in the mixed acid and stirred at room temperature for 6 h. After standing for 18 h, the mixture was washed several times with deionized water until the pH of the filtrate reached approximately 5. The acid-treated CNTs were finally obtained after drying.

#### 2.1.3. Preparation of Polyaniline-Coated Carbon Nanotubes (PANI-CNTs)

To synthesize polyaniline-coated carbon nanotubes (PANI-CNTs), decompression distilled aniline monomers were utilized as the PANI precursor. The theoretical targeted PANI content on the CNT surface was set at approximately 10 wt% based on the precursor feed ratio. This was achieved by dispersing 1.0 g of the functionalized CNTs in 100 mL of 2 M HCl (37 wt%) followed by ultrasonication for 2 h at room temperature. Subsequently, 0.1 mL of aniline monomer was slowly added under ultrasonication at 30 °C. To initiate the polymerization of aniline, approximately 20 mL of 0.2 M ammonium persulfate (APS) was slowly added to the mixture over 3 h in an ice bath at 2.5 °C with constant stirring. Polymerization was allowed to proceed for 24 h, with the temperature maintained at 2.5 °C throughout the process. The resulting dark suspension was filtered and washed alternately with deionized water and ethanol until the filtrate became colorless. After drying in an oven at 80 °C overnight, PANI-CNTs with 10 wt% PANI loading were obtained.

#### 2.1.4. Preparation of the Ni-N-CNT Catalyst

A total of 200 mg of PANI-CNTs and 0.15 mmol of NiSO_4_·6H_2_O were dissolved in approximately 80 mL of acetonitrile and stirred continuously for 12 h. The resulting precipitate was collected by filtration, washed several times with acetonitrile, and vacuum-dried at 80 °C overnight to remove residual solvents and obtain the precursor. Finally, the precursor was placed in a tubular furnace for carbonization. The entire thermal treatment was conducted under a 10% H_2_/N_2_ atmosphere, heating to 950 °C to ensure optimal graphitization and active nitrogen retention at a ramp rate of 5 °C/min and maintaining this temperature for 3 h to yield the Ni-N-CNT product.

#### 2.1.5. Preparation of the Pt/Ni-N-CNT Catalyst

A total of 5.2 mg of PtCl_4_ and 100 mg of Ni-N-CNT were added to 60 mL of deionized water, stirred for 12 h, filtered, and vacuum-dried at 80 °C. Subsequently, the product was placed in a tubular furnace at 300 °C for 1 h under an N_2_/H_2_ atmosphere at a ramp rate of 5 °C/min to obtain the final Pt/Ni-N-CNT catalyst.

#### 2.1.6. Preparation of the Pt/Ni-CNT Catalyst

A total of 200 mg of acid-treated CNTs, 0.15 mmol of NiSO_4_·6H_2_O, and 5.2 mg of PtCl_4_ were dissolved in approximately 80 mL of acetonitrile. After stirring for 12 h, the mixture was filtered, washed several times with acetonitrile, and vacuum-dried at 80 °C overnight to obtain the Pt/Ni-CNT precursor. This product was then carbonized in a tubular furnace at 950 °C for 3 h under N_2_/H_2_ (10% H_2_) atmosphere at a ramp rate of 5 °C/min. This reducing atmosphere was specifically selected not only to prevent oxidation but also to induce mild hydrogen etching of the carbon matrix, creating abundant defect sites and hierarchical pores favorable for subsequent metal anchoring. This process yielded the Ni-N-CNT product.

#### 2.1.7. Pt/C Catalyst

To systematically evaluate the catalytic activity of the synthesized Pt/Ni-N-CNT, a commercial 20 wt% Pt/C catalyst (Johnson Matthey, London, UK) was utilized as the standard reference. The commercial sample was evaluated under identical electrochemical conditions to establish a reliable baseline for the fabricated materials.

### 2.2. Physical Characterization

#### 2.2.1. Scanning Electron Microscopy (SEM)

In the field of materials analysis, SEM plays a crucial role in characterization due to its ultra-high spatial resolution. Its imaging mechanism relies on capturing the secondary electron signals excited by a high-energy electron beam and converting this microscopic information into visual images, thereby intuitively presenting the microscopic morphology and structural distribution of the catalyst surface. In this study, a ZEISS Sigma 300 scanning electron microscope (Oberkochen, Germany) was utilized as the analytical tool to conduct an in-depth analysis of the microscopic structure–activity relationships of a series of samples.

#### 2.2.2. Transmission Electron Microscopy (TEM)

Transmission electron microscopy (TEM) possesses extremely high resolution, making it a vital characterization method for probing deep internal information and atomic-scale configurations of materials. This technique utilizes the transmission signals carried by the electron beam penetrating ultra-thin samples for imaging, thereby revealing microscopic properties such as the crystal phase characteristics, nanoparticle distribution, and interplanar spacing of the materials. To further investigate the microstructures of the low-Pt-based oxygen reduction reaction (ORR) catalysts in this study, a Talos F200S transmission electron microscope (FEI, Morristown, NJ, USA) was employed to systematically observe and statistically analyze the nanoscale morphological features and particle sizes of the materials.

#### 2.2.3. X-Ray Photoelectron Spectroscopy (XPS)

The core mechanism of X-ray photoelectron spectroscopy (XPS) relies on the photoelectric effect. By capturing and analyzing the kinetic energy characteristics of photoelectrons emitted from the surface, it enables profound determination of the elemental composition, electronic state distribution, and chemical environment of the target material’s shallow surface layers. To accurately track the binding energy fluctuations and valence state evolutions of different components in the prepared catalysts, the surface chemical states in this study were primarily analyzed using a Thermo Scientific K-Alpha XPS system (Waltham, MA, USA).

#### 2.2.4. X-Ray Diffraction (XRD)

X-ray diffraction (XRD) technology utilizes the diffraction phenomenon generated by the interaction between X-rays and the crystal lattice to effectively reveal the internal crystalline state of materials. The 2θ angles and peak intensities of the characteristic diffraction peaks in the patterns directly map the phase composition, crystal configuration, and lattice plane arrangement of the samples, serving as a crucial basis for qualitative and quantitative material analysis. In this experiment, a Bruker D2 Phaser diffractometer (Bremen, Germany) was used to conduct phase detection on the synthesized samples of various components. The obtained data provided a physical characterization basis for the experimental patterns and catalytic mechanisms discussed herein.

#### 2.2.5. Inductively Coupled Plasma Optical Emission Spectroscopy (ICP-OES)

Inductively coupled plasma optical emission spectroscopy (ICP-OES) is an advanced technique that utilizes high-temperature plasma to excite the sample and performs quantitative elemental analysis based on the generated characteristic emission spectra. Because the specific wavelength light intensity emitted by the target element in its excited state shows a strictly directly proportional relationship with its actual concentration, this technique exhibits excellent sensitivity, outstanding resolution, and advantages in high-throughput sample processing for elemental determination. To precisely determine the actual loading amount of the noble metal platinum in the catalysts prepared in this study, an iCAP PRO spectrometer (Thermo Fisher, Waltham, MA, USA) was selected for quantitative testing, thereby ensuring extremely high accuracy and reliability of the compositional data.

#### 2.2.6. Specific Surface Area and Pore Size Distribution Analysis

To deeply investigate the pore structure characteristics of the samples, the nitrogen adsorption–desorption method was employed in this study to determine the specific surface areas of their micropores and mesopores. The experimental process was carried out using an ASAP 2460 fully automated analyzer manufactured by Micromeritics (Norcross, GA, USA). In specific data processing, based on the theoretical foundation of the Langmuir monolayer adsorption model, combined with the BET equation, mathematical fitting of the test data was performed to achieve data analysis of the material’s specific surface area and pore size distribution.

### 2.3. Electrochemical Measurements

#### 2.3.1. Working Electrode Preparation

A glassy carbon electrode (GCE) with a diameter of 5 mm was selected as the working electrode in this study. To obtain a smooth, scratch-free substrate, the electrode surface was pre-polished with Al_2_O_3_ polishing powder in a figure-eight trajectory for 30 cycles, followed by cleaning and drying. Furthermore, considering that the formulation, preparation process, and surface properties of the catalyst ink directly impact the test results, we strictly formulated the catalyst ink according to a predetermined ratio prior to the three-electrode system testing. A commercial 20 wt% Pt/C catalyst was also prepared using the identical procedure to serve as a control group. Finally, a micropipette was used to drop-cast the successfully prepared catalyst dispersion onto the treated electrode, ensuring that the droplet formed a complete covering layer on the electrode surface. After natural drying, the sample was ready for use as the working electrode.

#### 2.3.2. Cyclic Voltammetry (CV) Testing

Cyclic voltammetry (CV), as a core electrochemical analysis technique, is widely applied to investigate electrode process kinetics and the intrinsic properties of materials. In this study, a CHI760 electrochemical workstation (Chenhua, Shanghai, China) was utilized to conduct CV curve testing on catalysts synthesized via different processes, aiming to deeply analyze their redox behavior and the electrochemical characteristics of the electrode materials. The electrochemical measurement system primarily comprised a glassy carbon electrode modified with the catalyst (working electrode), an Ag/AgCl electrode (reference electrode), and a platinum electrode (counter electrode). During the measurement, a 0.1 mol/L HClO_4_ solution was used as the electrolyte, and electrolysis experiments were conducted under acidic and alkaline conditions to examine the electrochemical response of the catalysts. Polarization curves were recorded at a scan rate of 50 mV/s within the potential range of −0.2 to 1.0 V (vs. SCE). To ensure an oxygen-free testing environment, the electrolyte was saturated with N_2_ for 30 min prior to the initiation of all experiments.

#### 2.3.3. Oxygen Reduction Reaction (ORR) Performance Testing

The experiments were tested in an oxygen-saturated electrolyte medium. The instrument operating parameters were set as follows: a rotation speed of 1600 rpm and a constant linear sweep voltammetry (LSV) scan rate of 5 mV/s. After immersing the electrode into the solution and ensuring no bubbles were attached to its surface, the test was initiated. The potential scan direction was set from positive to negative. The system recorded the polarization curves of current versus potential. We primarily evaluated their catalytic activity during the ORR process by measuring and comparing the half-wave potential (E_1/2_) values of different samples. To evaluate the intrinsic ORR kinetics, the Tafel slopes were derived from the mass transport-corrected kinetic current density (jk), which was calculated using the equation jk = (j × jL)/(jL × − j), where j is the measured current density and jL is the limiting diffusion current density. To formulate the catalyst ink, 10 mg of the catalyst powder was ultrasonically dispersed in a mixture of 1400 μL deionized water, 1400 μL ethanol, and 200 μL of 5 wt% Nafion solution to form a homogeneous suspension. Subsequently, 10 μL of the respective ink was drop-casted onto the glassy carbon electrode (diameter: 5 mm). To ensure comparable film thickness and consistent mass transport conditions across different samples, the total catalyst mass loading on the electrode was kept identical for all samples.

#### 2.3.4. Catalyst Durability Testing

For the characterization of catalyst lifespan, studies generally rely on two key techniques, chronoamperometry and accelerated degradation testing (ADT), to quantify the evolution of performance over time. Through relevant electrochemical methods, the activity fluctuations and performance degradation mechanisms of electrode materials under prolonged working conditions can be detected. To evaluate the service life of the materials, accelerated durability tests are typically employed to simulate a long-term electrochemical environment. In this study, based on the cyclic voltammetry (CV) technique, 5000 continuous cycles were scanned within a fixed potential range at a scan rate of 100 mV/s. Subsequently, by comparing the ORR polarization (LSV) curves before the initiation of the accelerated durability test and after the completion of 5000 cycles, the electrochemical durability of the target catalyst was rapidly determined.

#### 2.3.5. Electrochemical Impedance Spectroscopy (EIS) Testing

To investigate the electrochemical reaction processes and interfacial characteristics on the catalyst surface, electrochemical impedance spectroscopy (EIS) testing was conducted in this study. Its core principle is to apply a small-amplitude alternating current (AC) signal to the testing system and record its response patterns. The actual measurements were performed in an O_2_-saturated electrolyte with an AC frequency range spanning from 0.01 Hz to 100 kHz. Subsequently, Nyquist plots were utilized to interpret the measured data: the starting position of the arc in the plot indicates the equivalent series resistance (Rs), which includes the solution and the electrode, while the charge transfer resistance (Rct) caused by the Faradaic process is determined by the diameter span of the semicircle.

To ensure the reproducibility of the reported results, all catalyst syntheses and electrochemical tests were performed in at least three independent replicates. The data presented in the Section 3, including potentials, current densities, and Tafel slopes, represent the average values obtained from these multiple trials, with an observed variance of less than 5%.

## 3. Results

### Physical Characterization

**Figure 1:**
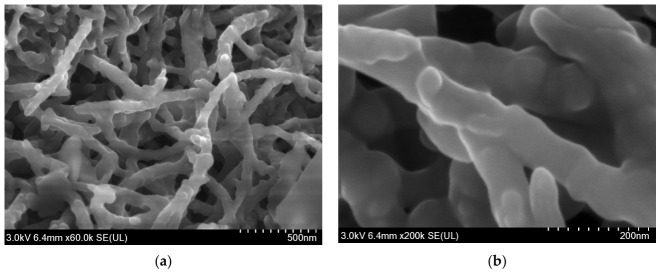

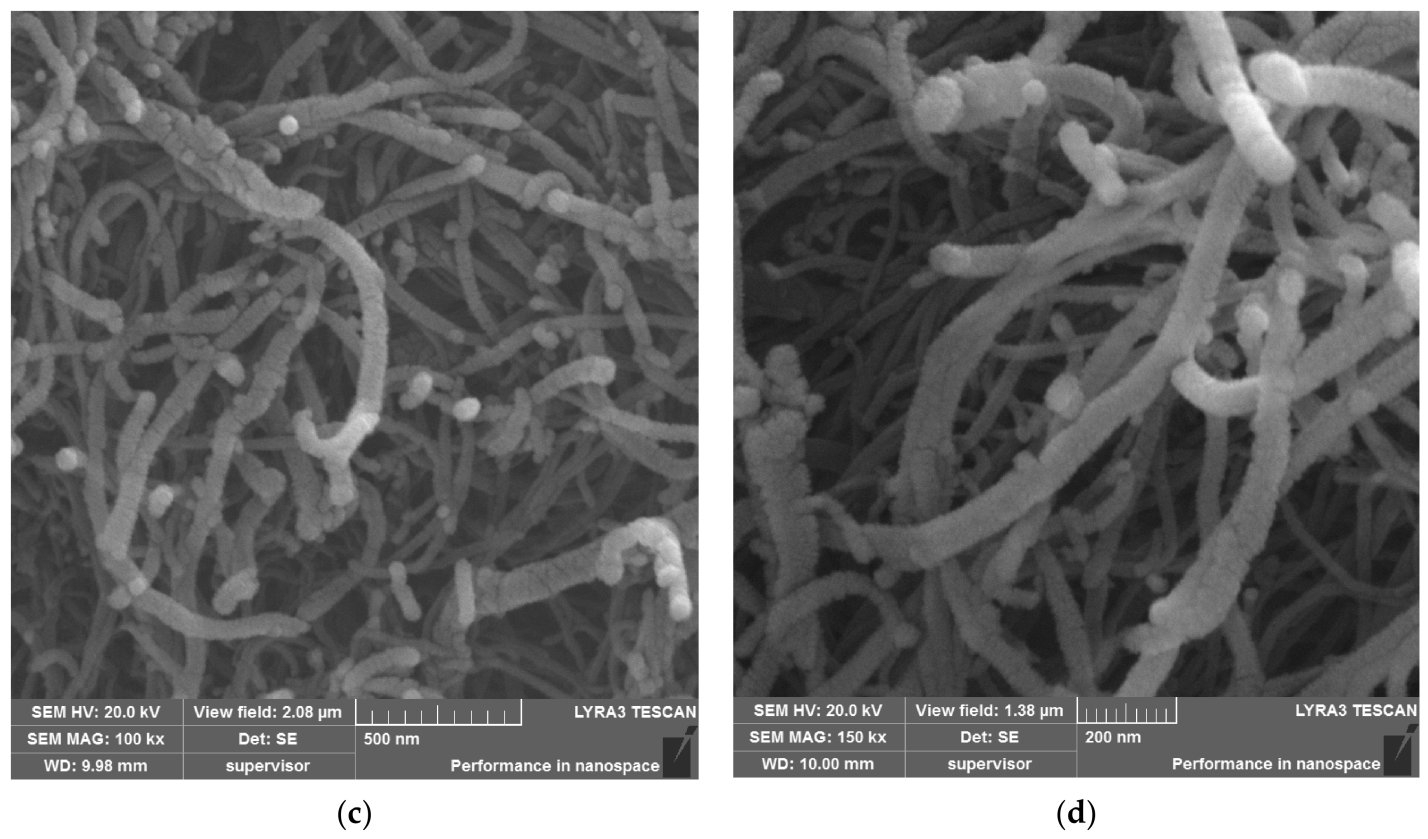
SEM images of Pt/Ni-N-CNT: (**a**) 500 nm; (**b**) 200 nm scale bars. SEM images of CNT: (**c**) 500 nm; (**d**) 200 nm.

Figure 1 displays the SEM images of the Pt/Ni-N-CNT catalyst. As revealed in Figure 1a, the catalyst preserves the intertwined three-dimensional (3D) network structure of the carbon nanotubes, a feature that is highly favorable for mass transport and charge transfer during the catalytic process. As shown in Figure 1b, in contrast to the typical diameter of pristine carbon nanotubes (Figure 1c,d), the observed nanotubes exhibit a significantly increased diameter (approximately 50–80 nm, as indicated by the 200 nm scale bar). This demonstrates the successful encapsulation or loading of the polyaniline-derived carbon layer and metal components onto the CNT surfaces, forming a coating layer with a relatively uniform thickness.

**Figure 2:**
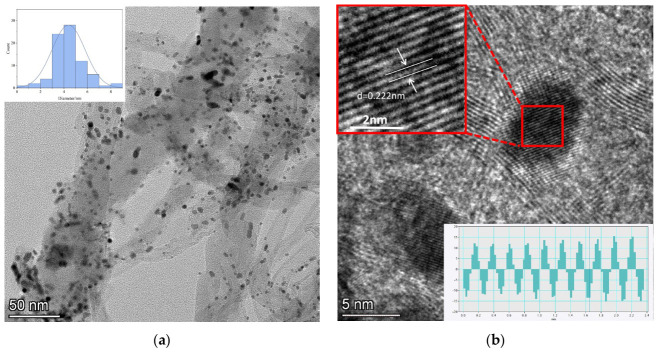
TEM images of Pt/Ni-N-CNT: (**a**) 50 nm; (**b**) 5 nm scale bars.

Figure 2 displays the TEM images of the Pt/Ni-N-CNT catalyst. As shown in Figure 2a, metal nanoparticles are highly dispersed on the walls of the nitrogen-doped carbon nanotubes, with no significant particle agglomeration observed. Furthermore, a rigorous statistical analysis of 79 individual Pt nanoparticles (Figure 2a) quantitatively confirms a narrow size distribution centered at approximately 5 nm, demonstrating the highly uniform dispersion achieved by our synthesis strategy. As illustrated in Figure 2b, the measured lattice spacing for the (111) crystal plane of Pt is 0.222 nm. Crucially, this value is smaller than the standard reference spacing of 0.226 nm for pure bulk Pt. This reduction in crystal plane suggests the presence of lattice contraction and compressive strain within the Pt nanoparticles, likely induced by strong interactions with the Ni-N-C support.

**Figure 3:**
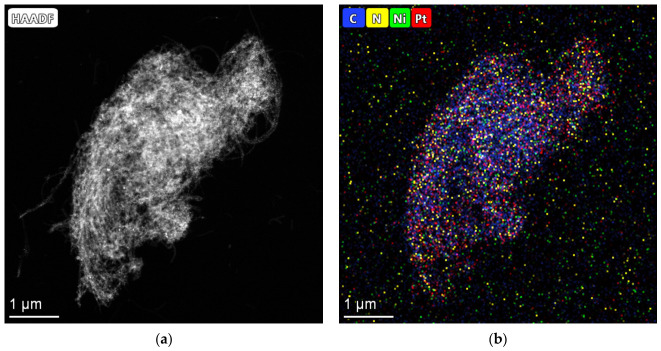

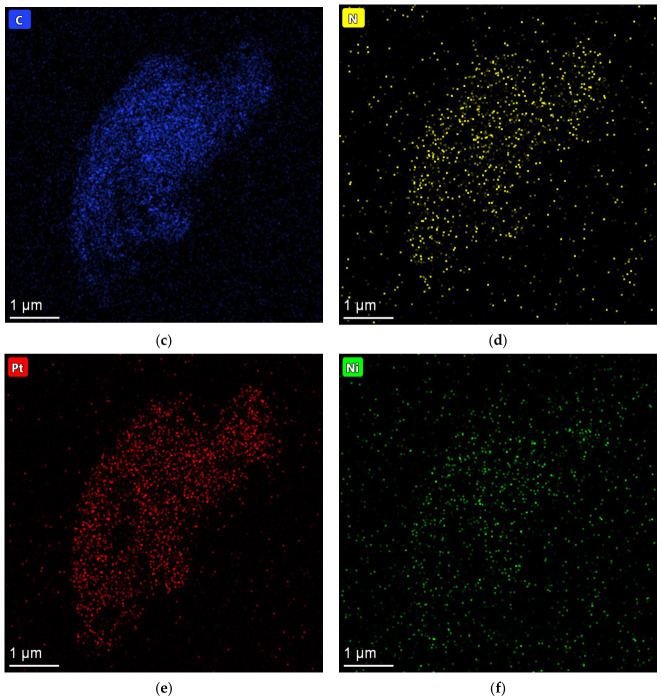
EDS mapping of Pt/Ni-N-CNT: (**a**) HAADF; (**b**) all elements; (**c**) C, (**d**) N, (**e**) Pt, and (**f**) Ni.

Figure 3 illustrates the energy-dispersive X-ray spectroscopy (EDS) elemental mapping analysis of Pt/Ni-N-CNT. As shown, the four elements—C, N, Pt, and Ni—exhibit highly overlapped and exceptionally uniform spatial distributions at the micrometer scale. Specifically, the near-complete overlap between the N and C elemental contours indicates that N is uniformly coated on the surface of the carbon nanotubes, which provides homogeneous sites for anchoring metal precursors. Importantly, no localized aggregation of Ni signals was detected. Combined with the HRTEM observations (Figure 2), where no distinct lattice fringes corresponding to metallic Ni or NiO clusters could be found, these results confirm that the Ni species do not form separate bulk phases. Instead, the Ni atoms are deep-seated and atomically integrated within the nitrogen-doped carbon matrix. To quantitatively complement the elemental mapping, the corresponding EDS compositional analysis (Table S2) reveals a substantial nitrogen content of 2.24 at%. These quantitative results unambiguously verify the successful and abundant incorporation of polyaniline-derived nitrogen species into the carbon framework, providing a chemically robust surface for subsequent metal anchoring. To accurately determine the bulk elemental composition of the Pt/Ni-N-CNT catalyst, inductively coupled plasma optical emission spectrometry (ICP-OES) was performed. As shown in Table S1, the mass fractions of Pt and Ni were determined to be 2.10 wt% and 0.05 wt%, respectively, corresponding to a Pt/Ni atomic ratio of approximately 12.6:1. This confirms that the catalyst achieves an ultra-low Pt loading.

**Figure 4:**
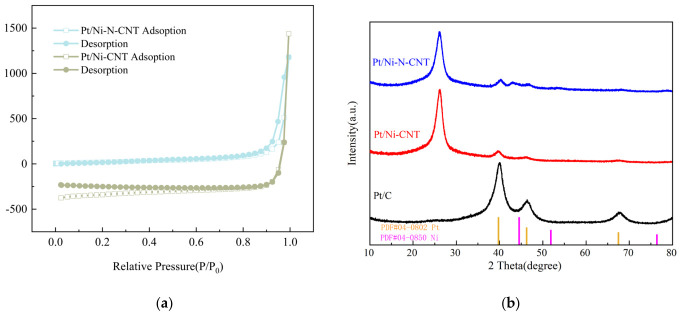
(**a**) N_2_ adsorption–desorption isotherms of various materials: Pt/Ni-N-CNT and Pt/Ni-CNT. (**b**) XRD patterns of Pt/Ni-N-CNT, Pt/Ni-CNT, and commercial Pt/C.

The pore structure characteristics of the catalysts were further investigated through N_2_ adsorption–desorption isotherms. As shown in Figure 4a, both Pt/Ni-N-CNT and Pt/Ni-CNT exhibit typical Type IV isotherms accompanied by H3-type hysteresis loops in the high relative pressure region. To further investigate the specific surface area and porous structure of the samples, N_2_ adsorption–desorption isotherms were collected (Figure 4). The quantitative analysis reveals that the Pt/Ni-N-CNT catalyst possesses a large specific BET surface area of 104.88 m^2^/g, which is notably higher than that of the non-doped Pt/Ni-CNT (95.79 m^2^/g). This indicates that the interior of the material is primarily composed of interconnected carbon nanotubes forming abundant slit-like pore structures, while the sharp rise in adsorption capacity at P/P_0_ > 0.9 confirms the presence of a large number of macropores. The open pore structure provides a more sufficient accessible surface area for active site exposure, and the abundant mesopores and macropores significantly facilitate charge transfer during the oxygen reduction process, thereby enhancing the overall electrochemical activity. As shown in Figure 4b, XRD patterns were employed to investigate the crystallographic structure of the synthesized catalysts. Pt/Ni-N-CNT and Pt/Ni-CNT exhibit a highly intense and sharp peak at ~26.0°, corresponding to the (002) plane of the highly graphitized carbon nanotube support. Furthermore, while the commercial Pt/C displays sharp diffraction peaks for crystalline Pt, the Pt (111) peak in Pt/Ni-N-CNT at ~40° is remarkably broad and weak. It should be noted that no explicit crystalline Ni or Ni-oxide peaks are detected in the XRD pattern. Furthermore, a broad shoulder is observed around 45.2°. This feature is primarily attributed to the shifted Pt(200) diffraction peak resulting from the significant lattice contraction upon Ni alloying. To quantitatively evaluate the degree of alloying, the lattice parameter was calculated based on Bragg’s law and the (111) diffraction peak. The calculated a for Pt/Ni-N-CNT is 0.3873 nm, which is smaller than that of pure Pt (0.3923 nm). Furthermore, according to Vegard’s law, the atomic fraction of Ni in the solid solution is estimated to be 12.5 at%, firmly establishing the successful formation of the Pt-Ni alloy and the resulting structural strain.

**Figure 5:**
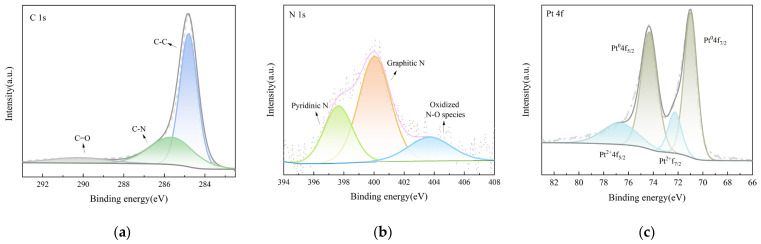
High-resolution XPS spectra of Pt/Ni-N-CNT: (**a**) C 1s, (**b**) N 1s, and (**c**) Pt 4f.

As illustrated in Figure 5, X-ray photoelectron spectroscopy (XPS) was further employed to investigate the surface chemical composition and electronic states of the Pt/Ni-N-CNT catalyst. All binding energies were rigorously calibrated by referencing the C 1s peak of adventitious carbon to 284.8 eV to eliminate any charging effects. As shown in Figure 5a, the C 1s spectrum of Pt/Ni-N-CNT exhibits three deconvoluted peaks at approximately 284.8, 285.8, and 289.0 eV, corresponding to C-C, C-N, and C=O bonds, respectively. As depicted in Figure 5b, the high-resolution N 1s spectrum of Pt/Ni-N-CNT reveals three characteristic peaks at approximately 398.5 eV (pyridinic N), 400.1 eV (graphitic N), and 403.8 eV (N-O species) [27]. To evaluate the effectiveness of targeted nitrogen doping, the surface composition was quantitatively analyzed. The XPS survey spectrum reveals a substantial overall nitrogen atomic fraction of 1.6 at%. More importantly, the deconvolution of the high-resolution N 1s spectrum (Table S3) demonstrates that pyridinic N constitutes the dominant fraction, accounting for 26.36% of the total nitrogen species. Among these, the abundant pyridinic N serves as strong coordination sites that facilitate the firm anchoring of ultra-low-loading Pt nanoparticles and suppress their agglomeration, while graphitic N effectively promotes the efficient four-electron ORR process. As shown in Figure 5c, the Pt 4f spectrum is primarily composed of metallic Pt0 and a minor portion of oxidized Pt^2+^, with peak positions located at Pt^0^ 4f_7/2_ (~71.1 eV), Pt^0^ 4f_5/2_ (~74.4 eV), Pt^2+^ f_7/2_ (~72.4 eV), and Pt^2+^ 4f_5/2_ (~75.8 eV). Compared to the Pt^0^ 4f_7/2_ peak of commercial Pt/C (typically situated at 71.2–71.4 eV), the binding energy of Pt/Ni-N-CNT exhibits a slight negative shift, which is primarily attributed to electron transfer from Ni or the nitrogen-rich carbon support to Pt, leading to an increased electron density on the Pt surface. Such electronic interactions between components effectively modulate the d-band center of Pt, inducing a moderate downward shift that weakens the strong adsorption of oxygen-containing intermediates (e.g., *OH) on the Pt surface, thereby significantly enhancing the ORR kinetic performance. Furthermore, the high-resolution Ni 2p XPS spectrum (Figure S1) exhibits a strong noise background with no identifiable characteristic peaks, resulting in an extremely low signal-to-noise ratio. This observation aligns well with our proposed structural model: because the Ni atoms are covered by the Pt layer, the limited probing depth of XPS leads to significant attenuation of the photoelectron signal.

**Figure 6:**
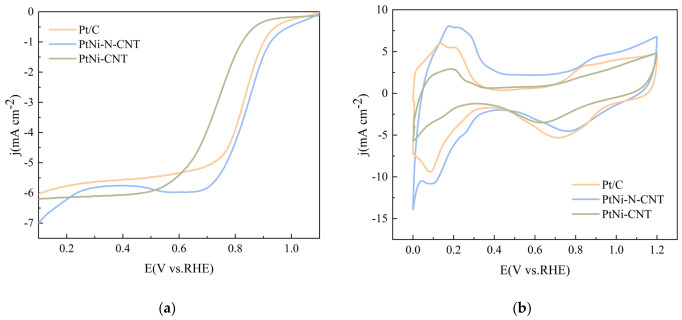

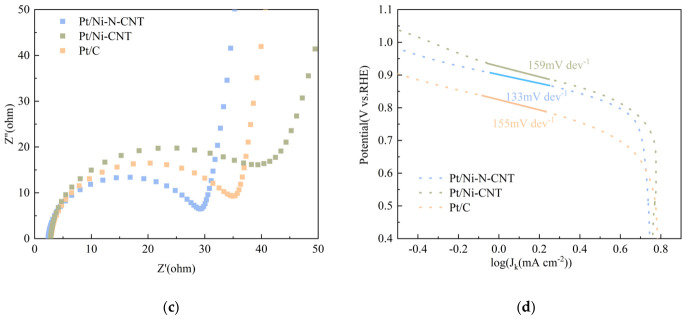
Electrochemical characterization of Pt/Ni-N-CNT, Pt/Ni-CNT, and commercial Pt/C: (**a**) LSV curves at 1600 rpm, (**b**) CV curves, (**c**) EIS Nyquist plots, and (**d**) Tafel plots (Note: The linear fitting for calculating the exact Tafel slopes was strictly performed in the high-potential, purely kinetically controlled region between 0.85 V and 0.95 V vs. RHE.).

The oxygen reduction reaction (ORR) performance of the Pt/Ni-N-CNT, Pt/Ni-CNT, and commercial Pt/C catalysts was investigated using a three-electrode system in an acidic electrolyte. Specifically, all ORR measurements were conducted in a 0.1 M HClO_4_ electrolyte. Prior to testing, the electrolyte was purged with high-purity O_2_ gas for at least 30 min to achieve steady-state saturation. The Linear Sweep Voltammetry (LSV) curves were recorded at a standard rotation speed of 1600 rpm with a scan rate of 0.05 mV/s. As shown in the LSV curves in Figure 6a, the Pt/Ni-N-CNT catalyst exhibits the highest ORR activity, with a half-wave potential (E_1/2_) of 0.846 V, which is superior to those of Pt/C (E_1/2_ = 0.81 V) and Pt/Ni-CNT (E_1/2_ = 0.72 V). To unambiguously distinguish the individual catalytic contributions of the structural components, a “blank” Ni-N-CNT support and a pure Pt-N-CNT control sample were systematically characterized (Figure S4). In the acidic electrolyte, the bare Ni-N-CNT exhibited extremely low ORR activity, confirming that the highly dispersed Pt nanoparticles act as the primary catalytic centers. More importantly, the pure Pt-N-CNT control delivered an E_1/2_ of only 0.81 V, which is significantly inferior to the target Pt/Ni-N-CNT catalyst (0.846 V). Figure 6b presents the CV curves, where Pt/Ni-N-CNT displays the largest hydrogen adsorption–desorption area in the potential range of 0–0.4 V (vs. RHE). Since the magnitude of the electrochemical active surface area (ECSA) reflects the number of exposed active sites, Pt/Ni-N-CNT possesses the highest ORR activity. To strictly evaluate the accessible active sites, the Electrochemically Active Surface Area (ECSA) of the catalysts was quantitatively calculated based on the hydrogen underpotential deposition (H_upd_) region in the CV curves (Figure S3). The Pt/Ni-N-CNT catalyst exhibits a remarkably high ECSA of 30.59 m^2^/g Pt, outperforming commercial Pt/C (25.9 m^2^/g Pt). The electrochemical impedance spectroscopy (EIS) results in Figure 6c show that Pt/Ni-N-CNT exhibits the lowest impedance. Furthermore, the ORR kinetics were analyzed using Tafel slopes; as shown in Figure 6d, the Tafel slope of Pt/Ni-N-CNT is 133 mV dec^−1^, which is significantly lower than those of Pt/C (155 mV dec^−1^) and Pt/Ni-CNT (159 mV dec^−1^), demonstrating the faster ORR kinetics of the Pt/Ni-N-CNT sample. As shown in Figure S2, to comprehensively elucidate the catalytic mechanism beyond E_1/2_, we have performed Koutecky–Levich (K-L) analysis to determine the reaction pathway. The K-L plots display excellent linearity and parallelism across various potentials, indicating stable first-order reaction kinetics. Importantly, the calculated electron transfer number (n) is approximately 3.69. This verifies that our ultra-low-loading Pt/Ni-N-CNT catalyst predominantly catalyzes the ORR via the highly efficient four-electron reduction pathway to water, directly confirming its superior intrinsic catalytic mechanism. To strictly evaluate the practical efficiency of the ultra-low-Pt-loading catalyst, the mass activity (MA) normalized by the actual Pt mass was calculated based on the kinetic current density at 0.85 V (vs. RHE). The MA of Pt/Ni-N-CNT reaches an impressive 0.696 A/mg Pt, higher than that of the commercial 20 wt% Pt/C catalyst (0.407 A/mg Pt). This outstanding mass-normalized performance confirms that the strong electronic interactions and uniform dispersion provided by the Ni-N-C support maximize the intrinsic turnover efficiency of every single Pt atom, successfully addressing the critical trade-off between cost and performance.

**Figure 7:**
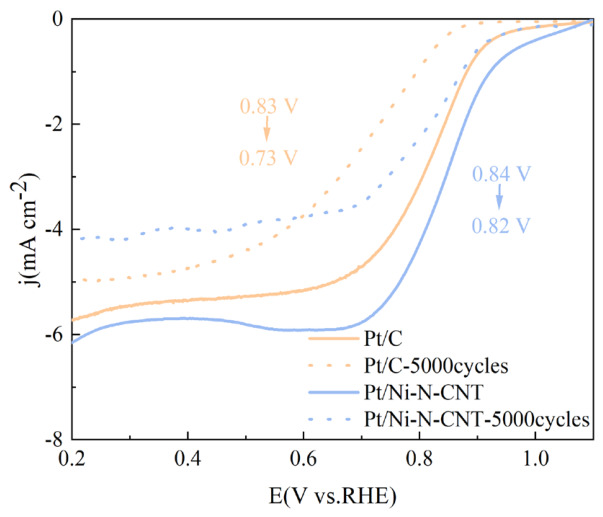
Electrochemical stability tests of Pt/Ni-N-CNT and commercial Pt/C catalysts after 5000 potential cycles.

To strictly quantify the degree of degradation, the shift in the half-wave potential (E_1/2_) was evaluated before and after the 5000-cycle stress test. As shown in Figure 7, the commercial Pt/C benchmark suffered a severe kinetic degradation, with its E_1/2_ dropping drastically by 80 mV (from 0.81 V to 0.73 V). In sharp contrast, the Pt/Ni-N-CNT catalyst exhibited significantly enhanced durability, maintaining robust ORR kinetics with a restricted E_1/2_ shift of only 26 mV (from 0.846 V to 0.82 V).

To further evaluate the performance of Pt/Ni-N-CNT in a broader context, we compared its ORR activity with other state-of-the-art catalysts reported recently (see Table S4 in Supplementary Materials [28,29]). While complex noble-metal alloys may exhibit higher potentials, our Pt/Ni-N-CNT delivers exceptional mass-normalized activity and robust stability at ultra-low Pt loadings, underscoring the practical potential of leveraging polyaniline-derived metal–support interactions.

## 4. Conclusions

To address the challenges of high noble metal consumption and mass transfer limitations in the oxygen reduction reaction (ORR), an ultra-low-platinum-loading Pt/Ni-N-CNT electrocatalyst was developed. Driven by the synergistic metal–support interactions and the robust N-doped carbon nanotube network, the electrochemical results demonstrate that, benefiting from these structural advantages, Pt/Ni-N-CNT exhibits exceptional ORR catalytic activity at ultra-low Pt loading, with both its half-wave potential (E_1/2_) and limiting diffusion current density surpassing those of commercial Pt/C, accompanied by rapid reaction kinetics. Furthermore, the catalyst demonstrates robust degradation resistance during 5000 continuous potential cycles. Furthermore, while the robust degradation resistance observed during the 5000-cycle accelerated durability test confirms the highly stable metal–support interactions at the half-cell level, the highly graphitized, hierarchical 3D nanotube network holds significant promise for mitigating carbon corrosion and managing complex mass transport in practical Membrane Electrode Assembly (MEA) fuel cell operations. Future work will focus on evaluating this catalyst layer in real fuel cell devices.

## Data Availability

The original contributions presented in this study are included in the article/Supplementary Material. Further inquiries can be directed to the corresponding author.

## Supplementary Materials

The following supporting information can be downloaded at: https://www.mdpi.com/article/10.3390/ma19112331/s1, Figure S1. High-resolution Ni 2p XPS spectrum of the Pt/Ni-N-CNT catalyst; Figure S2. (a) ORR polarization curves of Pt/Ni-N-CNT at various rotation speeds and (b) electron transfer umber (n); Figure S3. Quantitative comparison of the Electrochemically Active Surface Area (ECSA) for commercial Pt/C, the Pt/Ni-N-CNT catalys, calculated from the hydrogen adsorption-desorption charges in their respective cyclic voltammograms; Figure S4. LSV curves of Pt-N-CNT, Pt/Ni-CNT and Ni-N-CNT in 0.1 M HClO_4_ at 1600 rpm; Table S1. Quantitative bulk elemental composition and Pt/Ni atomic ratio of the Pt/Ni-N-CNT catalyst determined by ICP-OES analysis; Table S2: Quantitative elemental composition of the Pt/Ni-N-CNT catalyst derived from EDS analysis; Table S3: Quantitative surface composition of the Pt/Ni-N-CNT catalyst derived from XPS analysis; Table S4: Comparison of ORR Performance in Acidic Media.

## Abbreviations

The following abbreviations are used in this manuscript:

ORR	Oxygen Reduction Reaction
PANI	Polyaniline
CNT/CNTs	Carbon Nanotube(s)
SEM	Scanning Electron Microscopy
TEM	Transmission Electron Microscopy
XPS	X-ray Photoelectron Spectroscopy
BET	Brunauer–Emmett–Teller
LSV	Linear Sweep Voltammetry
CV	Cyclic Voltammetry
EIS	Electrochemical Impedance Spectroscopy
ADT	Accelerated Durability Test
