# Supplementary material for: Pt Catalysts Supported on Ni-N-Doped Carbon Nanotubes for Oxygen Reduction Reaction
Source: Materials (Basel). 2026 Jun 1;19(11):2331. doi: 10.3390/ma19112331 (PMC13258206; doi:10.3390/ma19112331)
Supplement: Supplementary file 1 [file materials-19-02331-s001.zip › materials-4284591-supplementary.pdf]

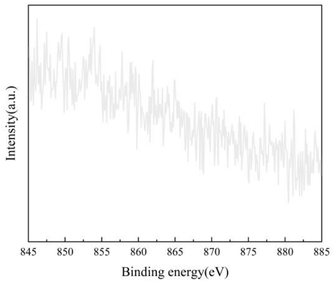

Figure S1. High-resolution Ni 2p XPS spectrum of the Pt/Ni-N-CNT catalyst.

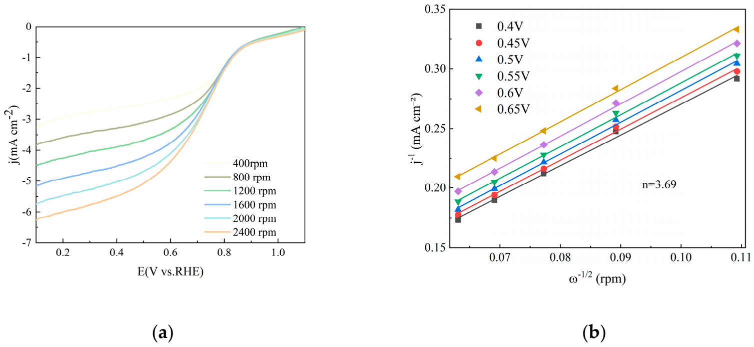

Figure S2 (a) ORR polarization curves of Pt/Ni-N-CNT at various rotation speeds and (b) electron transfer umber (n).

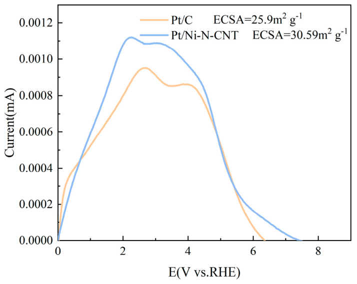

Figure S3. Quantitative comparison of the Electrochemically Active Surface Area (ECSA) for commercial Pt/C, the Pt/Ni-N-CNT catalys, calculated from the hydrogen adsorption-desorption charges in their respective cyclic voltammograms.

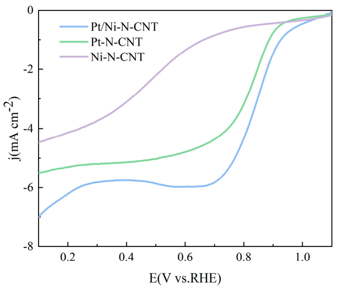

Figure S4. LSV curves of Pt-N-CNT, Pt/Ni-CNT and Ni-N-CNT in 0.1 M $HClO_4$ at 1600 rpm.

Table S1. Quantitative bulk elemental composition and Pt/Ni atomic ratio of the Pt/Ni-N-CNT catalyst determined by ICP-OES analysis.

Element Tag	weight g	volume mll	Instrument reading mg/L	Sample concentratio n	unit	mass percent%
Ni	0.021	50	0.2099	499.7288	mg/kg	0.0500
Pt	0.021	50	8.815	20988.0573	mg/kg	2.0988

Table S2: Quantitative elemental composition of the Pt/Ni-N-CNT catalyst derived from EDS analysis

Element	Atomic Fraction (at%)	Mass Fraction (wt%)
C (Carbon)	97.25	91.63
N (Nitrogen)	2.24	2.47
Ni (Nickel)	0.17	0.76
Pt (Platinum)	0.34	5.14
Total	100.00	100.00

Table S3: Quantitative surface composition of the Pt/Ni-N-CNT catalyst derived from XPS analysis.

Element	Overall Surface Atomic Fraction (at%)	Nitrogen Species	Binding Energy (eV)	Relative Proportion (%)
C 1s	91.81 at%	-	-	-
		Pyridinic N	~398.5	26.36 %
N 1s	1.6 at%	N-O species	~400.0	19.16%
		Graphitic N	~401.0	54.58 %

Ni 2p	0.11 at%	-	-	-
Pt 4f	0.48 at%	-	-	-

Table S4: Comparison of ORR Performance in Acidic Media

Catalyst	Pt Loading (wt%)	$E_{1/2}$ (V vs. RHE)	Tafel Slope (mV/dec)	Reference
Pt/Ni-N-CNT	Ultra-low (5.2 mg precursor)	0.846	133	This work
Commercial Pt/C	20%	0.81	155	This work
Pt-Ni Nanoframes	5-10%	~0.90	~70	Ref [~~24~~28]
Pt/N-C Single Atom	< 5%	0.85-0.88	80-100	Ref [~~25~~29]
Pt/CNT(Acid treated)	Ultra-low	0.72	159	This work

Commented [M1]: Please specify the detailed reference information.

Commented [1R2]: I have updated the references in the supplementary information.
